# Innovating the practice of medical speciality training

**DOI:** 10.1007/s40037-015-0245-1

**Published:** 2016-01-11

**Authors:** Joanne P. I. Fokkema

**Affiliations:** Tweede Oosterparkstraat 249, 1092 BM Amsterdam, The Netherlands

**Keywords:** Medical speciality training, Postgraduate training, Educational innovations, Effects of innovations

## Abstract

Educational innovations are being introduced into medical speciality training. But how do people who participate in medical speciality training (residents, consultants, programme directors) deal with these innovations? And what effects do educational innovations have according to these people?

By addressing these questions, this thesis contributes to the knowledge about the challenging process of innovating medical speciality training.

## Introduction

Educational innovations are being introduced into medical speciality training in response to changes in healthcare demands and related changes in requirements regarding physicians’ performance [1]. Many recent innovations in speciality training, such as workplace-based assessment (WBA), are related to the contemporary view that physicians’ training should be competency based and outcome oriented [2]. Medical education research up until now has mainly focused on the intended educational effects of innovative methods such as WBA [3]. However, bringing about the intended changes in practice turns out to be challenging and largely dependent on how innovations are handled by the people involved [4]. It is still largely unclear what is needed for people in daily practice to pick up educational innovations in a meaningful way. With this thesis, we aimed to contribute to the knowledge about the challenging process of innovating medical speciality training.

We drew upon literature from business, social psychology, sociology and healthcare, and focused on the experiences of the people who participate in medical speciality training: residency programme directors, consultants, and residents. The overall question this thesis addressed was: how do people who participate in medical speciality training deal with implementing and using innovations in this training?

## Methods

We started by looking into three distinct aspects that are involved with innovation: approaches of the people in charge of training for bringing about change, the effects in practice that using an innovation brings about, and the perceptions of the people involved with training regarding these effects. These elements were combined in a case study of an innovation process. We conducted these studies with a constructivist epistemology, meaning that we assume that knowledge about the phenomenon of study is constructed in dialogue between researchers and participants [5].

Our first study was a qualitative exploration of programme directors’ approaches to change at a training department using semi-structured interviews with a purposeful sample of 16 programme directors from different specialities. Its design was based on notions from corporate business and social psychology about the roles of change managers. The second study was an exploratory qualitative study for establishing what types of effects of an innovation its users perceive. It focussed on WBA as a case of an innovation in speciality training that is widely known and used. We conducted semi-structured interviews with 17 purposively sampled Dutch trainees (*N* = 7) and (lead) consultants (*N* = 10) in surgical and nonsurgical specialities. To encourage exploration of effects outside the domain of education, the study design was informed by sociological theory on the diffusion of innovations. In these first two studies, interview transcripts were analyzed thematically using template analysis. Our third study was a Q methodological study aiming to explore the distinct perceptions that users of the same innovation can have about its effects, again focussing on WBA as a case. Purposively sampled obstetrics–gynaecology residents (*N* = 22) and attending physicians (*N* = 43) at six hospitals in the Netherlands performed individual Q sorts by ranking 36 statements concerning the method of WBA and WBA tools according to their level of agreement. By-person factor analysis was conducted to uncover patterns in the ranking of statements, followed by interpretation using participant comments about their Q sorts. Our final study aimed to gain insight into effects of an innovation and how these are influenced by the implementation approach. For this purpose, a case study was conducted of a Dutch project that aimed to improve accountability and quality of speciality training. Using a theory-driven methodology based on a general theory of implementation [6], proceedings of project meetings were thematically analyzed to identify choices and developments regarding the implementation approach and to assess the effects of various approaches.

## Results

In the first study we found that lead consultants approach educational change using idiosyncratic change strategies [7]: they had individual ideas and beliefs about change that clearly influenced what they regarded as the best way to manage change. They differed in their degree of awareness of the strategies they used, and in the way in which they reflected on their efforts. Differences in knowledge, task interpretation, and personal style also influenced their approaches, as did culture and customs in the department.

Our second study illustrated that an innovation can bring about a variety of effects that extend beyond the range of the intended, expected, and desired effects [8]. Trainees and consultants experienced effects of WBA in six domains of their professional lives: *sentiments (affinity with the innovation* and *emotions), dealing with the innovation, speciality training, teaching and learning, workload and tasks*, and *patient care*. Affinity with the innovation varied between users and appeared to be one of the influences on *teaching and learning* effects. Organizational support and the match between the innovation and practice were considered important to minimize additional workload and to ensure that the WBA was used in a way that is relevant for training. Dealing with WBA stimulated attention for speciality training and placed speciality training on the agenda of clinical departments.

In the Q study to determine perceptions of trainees and consultants regarding the effects of using WBA, we found five distinct perception profiles: *enthusiasm, compliance, effort, neutrality*, and *scepticism* [9]. The five perceptions were characterized by differences in views on three main issues: the goals the innovation was intended to achieve, its applicability in practice, and its actual impact. Thus, we found that those involved in an innovation can vary substantially in their perceptions of effects of that innovation, even if they work in the same department and have similar characteristics such as function or amount of experience.

The final study used a Dutch innovation process to improve accountability and quality of speciality training as a case. This innovative project included facilitating openness of information about all speciality training programmes, including quality indicators and ratings of these programmes, and stimulating development of new programmes. The overarching finding was that effects of the innovation and features of the implementation approach were strongly intertwined. For instance, the implementation approach depended on appealing to the professional pride of programme directors, but was supplemented by pressure to participate. This did elicit use of the innovation by parties who had first resisted, but in a way that was not conducive to the goals. Furthermore, the approach involved stakeholder involvement. This revealed obstacles for implementation, to which the approach was then adapted. Attending to these obstacles enabled the development of effects that went beyond the intentions, but that were relevant for training quality (e.g. growing awareness of modern training principles).

## Discussion

The answer to our main research question ‘how do people who participate in medical speciality training deal with innovations in this training?’ consists of several components. The people involved in speciality training deal with innovations in a variety of ways and consequently innovations lead to a range of effects, which are again perceived in various ways. People’s approaches to innovations depend on interplay between different factors. Affinity with the innovation was uncovered as being among the most prominent. Furthermore, other personal factors such as individual ideas, beliefs and understanding of the innovation, and social and contextual factors (does it fit with routine practice) were of influence.

**Fig. 1:**
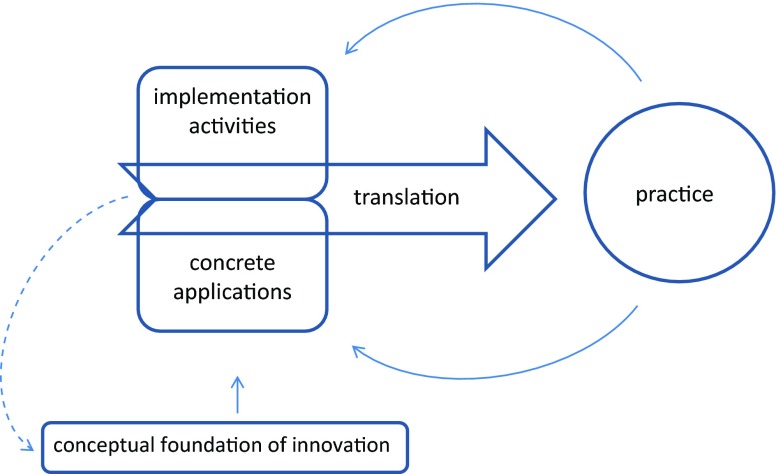
Dynamic model representing the proposed new perspective on innovating medical speciality training

The above-described complexity points to the need for a new conceptualization of innovating speciality training. We propose to abandon the dominant linear perspective where optimal implementation of an innovation will lead to certain intended effects, and suggest a more dynamic model that can account for the complexities of innovating speciality training (Fig. 1). The model links the *conceptual foundation* of the innovation, its *translation* to practice, and *effects* in practice. It accentuates the notion of *translation* of a concept to practice, which is formed by the combination of applications and implementation approach. This proposition to acknowledge complexity of innovation processes is consistent with a current shift in emphasis in implementation science research about innovations for healthcare [10, 11].

Our findings and the proposed conceptualization of innovating speciality training implicate a shift in focus for both research and practice; from innovative applications to the translation of innovative concepts that includes implementation approach. For medical education research, this entails the challenge of taking up methodologies that are fit to study complexity (e.g. reflexive monitoring in action). For practice, it means that training programmes of the innovation professionals of the future, as in educational or management studies, need to cover a broad terrain. Furthermore, all involved in innovating speciality training need to be aware of the complexity of the process as a basis for an appropriate approach. The insight provided by this thesis can prevent expecting unambiguity, clear-cut use of applications, and immediately reaching intended effects. In this way, this thesis supports realistic expectations and approaches for innovating the practice of medical speciality training.

### Tip

Writing is a crucial part of your PhD trajectory. Try to see it as an artful craft. Get a sense of what you want to tell about your research and then don’t postpone, start writing! I know it’s scary, but ask for feedback at an early stage. Determine what type of feedback you are seeking; do you need to shape your thoughts by discussing your results with colleagues, get directions from your supervisor or another expert, or is it style advice or confidence that a friend could provide?
